# Mucosal Adjuvants Delivered by a Mucoadhesive Patch for Sublingual Administration of Subunit Vaccines

**DOI:** 10.3390/ijms232113440

**Published:** 2022-11-03

**Authors:** Claire Monge, Camille Ayad, Anne-Lise Paris, Renaud Rovera, Evelyne Colomb, Bernard Verrier

**Affiliations:** UMR 5305: Laboratoire de Biologie Tissulaire et d’Ingénierie Thérapeutique, Institut de Biologie et Chimie des Protéines, CNRS/Université Claude Bernard Lyon 1, 7 Passage du Vercors, 69007 Lyon, France

**Keywords:** sublingual, adjuvant, nanoparticle, mucoadhesive, cytokine profiling, layer by layer, mucosal vaccine

## Abstract

Among mucosal administration routes for vaccines, the sublingual route has been proven capable of inducing a potent systemic and mucosal immune response. However, the absence of a simple and compliant delivery system and the lack of robust mucosal adjuvants impede the development of sublingual vaccines. Here, we describe a mucoadhesive patch made of a layer-by-layer assembly of polysaccharides, chitosan, and hyaluronic acid. The mucoadhesive patch was covered by adjuvanted nanoparticles carrying viral proteins. We showed that the nanoparticles effectively cross the outer layers of the sublingual mucosa to reach the epithelium. Furthermore, the encapsulated adjuvants, 3M-052 and mifamurtide, targeting toll-like receptor (TLR) 7/8 and nucleotide-binding oligomerization domain-2 (NOD2), respectively, remain fully active after encapsulation into nanoparticles and exhibit a cytokine/chemokine signature similar to the mucosal gold-standard adjuvant, the cholera toxin. However, the particulate adjuvants induced more moderate levels of proinflammatory interleukin (IL)-6 and keratinocyte chemoattractant (KC), suggesting a controlled activation of the innate immune response.

## 1. Introduction

Although mucosal vaccines are powerful tools for induction of mucosal immunity [1], there are still only a few on the market, owing to several technological and biological hurdles impeding the development of efficient formulations [2]. Among mucosal administration sites, sublingual (SL) mucosa presents an accessible and thin epithelium that has exhibited considerable potential to induce efficient vaccine response in non-human primates [3,4]. This administration route presents the advantages of compliance, needle-free application, and induction of both systemic and mucosal immune responses. The induction of a potent mucosal response is one of the main challenges to address to efficiently fight mucosal viral infections, as the secretion of mucosal IgA antibodies could protect both from infection and transmission [5,6]. Two main challenges have to be overcome for the development of a successful SL vaccine: (1) the design of an efficient mucoadhesive delivery system able to avoid dilution of the vaccine in the saliva and (2) immunogenic tolerance by the use of an effective adjuvant [7,8].

SL vaccines have shown an enhanced mucosal response when administered by needle-free injectors [4] or hydrogels [9], highlighting the potential of this administration route to activate mucosal immunity. However, simple, mucoadhesive, and compliant delivery systems still have to be designed. Sublingual patches composed of polysaccharides (chitosan, CHI, and hyaluronic acid (HyA)) assembled by layer-by-layer (LbL) technology have shown an enhanced retention time at the sublingual site and can be used as a mucoadhesive delivery platform [10].

Vaccine formulations administered by the SL route must be compatible with the tolerogenic environment of the buccal mucosa. The development of potent mucosal vaccines is a key issue in the design of SL vaccines. The role of a vaccine adjuvant is to improve the induced adaptive immune response by activating innate immune pathways. Innate immunity can be activated by the recognition of bacterial or viral components that activate specific pattern recognition receptors (PRRs) such as toll-like receptors (TLRs) or nucleotide-binding oligomerization domain (NOD) receptors present on the immune cell surface or in endosomes (for a review, see [11]). The stimulation of these sensors induces multiple immune signaling, leading to the activation and migration of antigen-presenting cells (APCs) to the draining lymph nodes, resulting in antibody and cell responses. Among the innate signaling responses, the induction of proinflammatory cytokines and inflammatory chemokines plays a central role to orchestrate immune cell activation, migration, and differentiation. Profiling of cytokine and chemokine expression patterns in response to vaccines is often associated with gene expression analysis and profiling of cell types and subsets to understand the innate mechanisms of mRNA vaccines in a systems vaccinology approach [12].

Originally, almost all studies of SL vaccination were based on the preclinical mucosal gold = standard adjuvant for SL vaccines, cholera toxin (CT), which is not suitable for human use. Therefore, another enterotoxin-based adjuvant candidate was developed, the double LT mutant (dmLT or R192G/L211A) [13], which has shown promising enhancement of mucosally induced immune response [4,14]. However, toxin-based adjuvants have a fragile tertiary structure, which complicates the production process and could denature the protein adjuvants. Other adjuvants have been evaluated for SL administration, such as alpha-galactosylceramide (GalCer), CpG-containing synthetic oligodeoxynucleotides (CpG-ODN), and cyclic di-adenosine monophosphate (c-di-AMP) [15,16]. However, because none of these candidates have been evaluated beyond preclinical studies, there is still a need to develop safe and effective adjuvants for clinical use that can induce potent systemic and mucosal immune responses after SL administration, together with an innovative mucosal delivery system. 

We previously shoed that the LbL mucoadhesive patch is an efficient platform for the delivery of proteins [10]; however, because the patch is not adapted to the delivery of hydrophobic adjuvant molecules, we developed a nanovaccine adsorbed on the surface of an LbL mucoadhesive patch. Here, we propose a mucoadhesive system for the delivery of a protein antigen (HIV-1 p24 antigen) absorbed on an adjuvanted particle. Two adjuvants were studied and compared to CT, the TLR 7/8 agonist telratolimod (3M-052) and the Nod2 agonist mifamurtide. 3M-052 is a member of the family of 3M imidazoquinoline immune response modifiers (IRMs) that stimulate innate immune responses through TLR7 and/or TLR8 [17]. 3M-052 is structurally similar to resiquimod (R848) but presents a higher hydrophobicity and improved bioavailability at the immunization site and the draining lymph nodes, owing to sustained release properties [18]. Mifamurtide is a derivative of muramyl dipeptide (MDP) and presents the same immunomodulatory properties with a longer half-life in plasma [19].

The adjuvants were incorporated into a poly(lactic acid) (PLA) nanoparticle (NP) before the adsorption of a model protein antigen from HIV-1, p24. Previous studies by our group highlighted the potential of encapsulated Nod2 agonist (murabutide) to induce a mucosal immune response after nasal administration [20]. The encapsulation of 3M-052 in poly(lactic glycolic acid) (PLGA) NP was described by others [21]. However, the effect of these particulate formulations of adjuvants administered by the SL route on innate immunity has not been explored to. 

In this work, we describe the development of a mucosal delivery system for the sublingual administration of an adjuvanted subunit nanovaccine consisting of a polymeric particle carrying both adjuvant and antigen. The encapsulation efficiency was assessed by dynamic light scattering (DLS), and the bioactivity of the encapsulated adjuvants was evaluated through the activation of TLR7/8 and Nod2 receptors in HEK reporter cells. Then, the effective uptake of NP by dendritic cells (DCs) was observed by confocal microscopy, in addition to an evaluation of NP in vivo transport through the SL mucosa of mice. Finally, the early activation of innate response was evaluated by the proinflammatory signature of the adjuvants and characterized by the quantification of cytokine levels in the serum after immunization with p24 adsorbed on adjuvanted NP.

## 2. Results and Discussion

### 2.1. Mucoadhesive SL Formulation for NP Delivery

Mucosal nanoparticle delivery was performed by presentation through a previously described mucoadhesive freestanding membrane (sublingual LbL patch) [10]. The mucoadhesive LbL patch was designed to strongly adhere to the buccal mucosa and release its cargo during the salivary degradation process.

**Figure 1:**
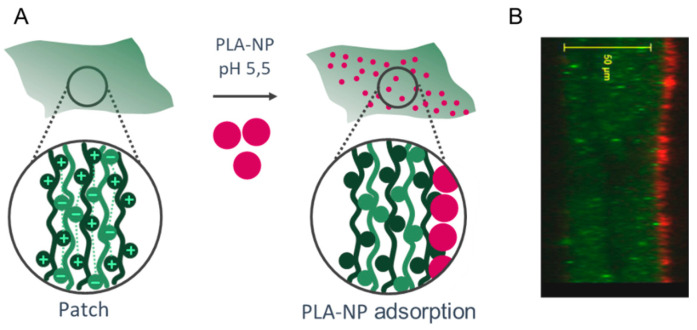
Mucosal NP delivery by a mucoadhesive LbL patch. (**A**) (CHI/HyA)_100_ patch and PLA-NP adsorption on the surface. (**B**) Optical section of the PLA-NP (bodipyTR, red) adsorbed on the mucoadhesive LbL patch (CHI^FITC^, green).

The NPs were adsorbed on the surface of the LbL patch mainly by electrostatic interactions of the positive charges of the last layer (CHI) with the negative charges of the PLA-NP (Figure 1). The presentation of PLA-NP on the surface is expected to induce a rapid release through the sublingual mucosa. The release of NP either relies on (1) pH variation from 5.5 (production) to 6.5 (sublingual pH), which loosens the electrostatic interactions between CHI and negative polymers (HyA and PLA), or (2) the enzymatic degradation by hyaluronidases, lysozymes, and amylases. 

### 2.2. Cytotoxicity

The PLA polymer used for NP production is considered a biodegradable, biocompatible, and safe polymer in various animal models and has been approved by the US FDA [22,23]. However, when formulated as an NP, the concentration of NPs used in the cell culture is of prime importance, as toxicity is associated with the NP density (i.e., the amount of NPs/cell). In our case, we determined that the PLA-NP can be delivered at a density of 10^4^ NPs/cell without inducing cytotoxicity.

**Figure 2:**
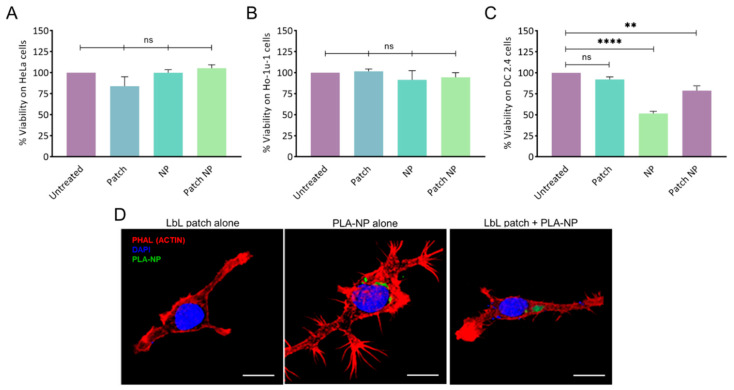
Cytotoxicity of NPs and the LbL patch on (**A**) human epithelial cells (HeLa), (**B**) human buccal epithelial cells (Ho-1u-1), and (**C**) murine dendritic cells (DC2.4). The percentage of viability was normalized to untreated cells. Data are presented as mean ± SD; statistical analysis was performed using one-way ANOVA followed by Tukey’s multiple comparison test (not significant (ns): *p* > 0.05; **: *p* < 0.001, and ****: *p* < 0.00001). (**D**) PLA-NP uptake by DC2.4 cells after release by the LbL patch. The LbL patches were degraded for 24h in artificial saliva before addition to cells. Actin (phalloidin-TRITC) and nucleus (DAPI) labelling were performed 20 min after incubation with an LbL patch, PLA-NP, or the combination thereof. NP internalization was observed using fluorescein–labelled PLA-NP. 3D reconstructions of optical sections were obtained from pictures taken by confocal microscopy. Scale bar: 10 µm.

The absence of cytotoxicity of the LbL patch, PLA-NP, and the combination thereof was confirmed on human epithelial cells (HeLa) and human buccal epithelial cells (Ho-1u-1) (Figure 2A,B). 

To assess the potential of the LbL patch to protect DC cells from the toxicity of a high NP concentration, 10^5^ NPs/cell were delivered directly or after adsorption/release from a patch degraded in artificial saliva (Figure 2C). The direct addition of NP onto the DC2.4 cell culture led to approximately 50% cell death, whereas the delivery of NPs from the patch increased the viability to 80%. This effect might be the result of the presence of a polysaccharide corona around the NP, limiting the overactivation of the endocytic process of the DCs.

### 2.3. NP Release from the LbL Patch and Uptake by Immune Cells 

Given the hypothesis that the PLA-NPs released from the LbL patch could be decorated by polysaccharides, possibly impeding PLA-NP endocytosis, the uptake of PLA-NPs by dendritic cells after release from the LbL patch was assessed. The PLA-NPs released from the LbL patch after 24 h of incubation in artificial saliva were incubated for 20 min with DC2.4 in culture. After observing that the presence of salivary enzymes did not affect NP uptake, we confirmed that the PLA-NPs released from the LbL patch were taken up by DCs to initiate the immune response (Figure 2D).

### 2.4. PLA-NP Transport across Sublingual Mucosa

The distribution of PLA-NP in the mouse oral cavity was followed by the administration of either a drop of 10 µL under the tongue or the deposition of the LbL patch containing the same quantity of PLA-NPs.

**Figure 3:**
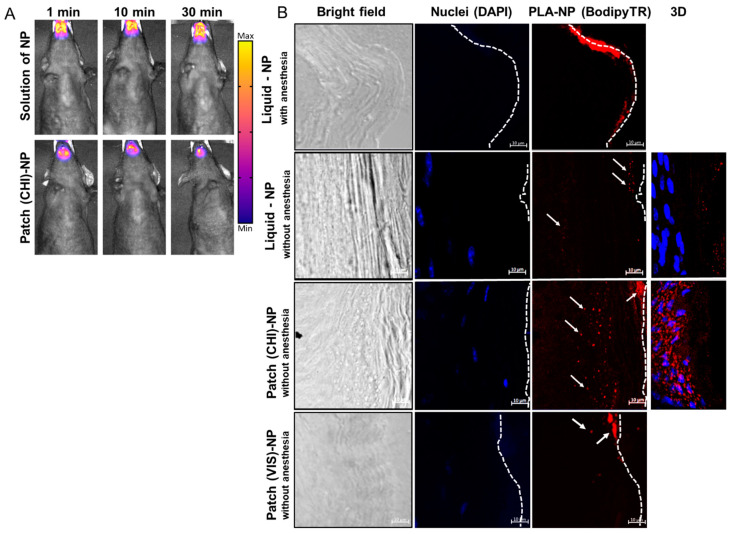
PLA-NP residence and transport through the SL mucosa. (**A**) Fluorescence molecular tomography of PLA-NP^ICG^ (indocyanine green) in solution or incorporated in the LbL patch. Fluorescent signal was detected 1, 10, or 30 min after administration. (**B**) PLA-NP visualized by confocal microscopy 10 min after administration as a liquid dosage or by a mucoadhesive LbL patch made of CHI or VIS combined with HyA. The pictures were taken with a 40× objective lens with a zoom factor of 4×. White arrows indicate fluorescent PLA-NPs, and dashed lines represent the outer surface of the SL mucosa. The panels on the right side of the figure are 3D reconstructions (maximum-intensity projection) of optical sections from 40 µm thick cuts of SL mucosa (ventral part of the tongue).

Live imaging experiments highlighted a similar residence time of the PLA-NPs in solution (10 µL under the tongue) or on the LbL patch of approximately 30 min for both dosages (Figure 3A). This could be explained by the use of gaseous anesthesia throughout the imaging process, avoiding the swallowing of the liquid formulation. 

Results of PLA-NP detection in the SL mucosa by confocal microscopy differed significantly anesthetized vs. unanesthetised animals (Figure 3B). The absence of post-administration anesthesia led to a drastic decrease in the amount of PLA-NPs accumulated on the mucosal surface, confirming that the SL administration of liquid formulations should be performed under prolonged anesthesia. This discrepancy can be explained by the probable dilution of liquid suspension in the saliva of awake animals and subsequent partial swallowing or by the penetration of the NPs by other buccal mucosal sites, such as checks, lips, gingiva, or palate. 

PLA-NPs released from the patch 10 min after application on the mouse SL mucosa already crossed the keratinized layer of the mucosa to reach the epithelium (nucleated cell) (Figure 3B). The efficient passage of PLA-NPs delivered by the LbL patch through the SL mucosa opened the possibility of using them as a mucosal nanovector. The presence of protein antigen p24 on the surface of the PLA-NPs did not influence the transport kinetics of the PLA-NPs.

Classical SL administration of liquid vaccines includes (1) a maximum volume of around 10 µL, (2) gaseous or injected anesthesia (intraperitoneal injection of ketamine/xylazine or analogs), and (3) a post-administration posture in anteflexion to avoid swallowing of the suspension [3,9,16]. Despite these precautions to avoid the swallowing of the vaccine, pre-clinical studies in non-human primates highlighted the lack of homogeneity in the induced immune responses after SL administration of a liquid formulation [24]. In our study, the animals were only lightly anaesthetized a few minutes before patch administration and were immediately free to swallow and groom a few seconds after administration. Figure 3B demonstrates the importance of the development of compliant SL administration systems for the clinical translation of buccal vaccination, especially for younger populations that are not expected to be collaborative during SL administration of a vaccine.

In a study by Masek et al., a mucoadhesive fibrous scaffold was developed to deliver polymeric and lipid-based NPs [25]. The SL administration of polymeric NPs by mucoadhesive scaffolds in piglets led to penetration of the particles and their transport to regional lymph nodes. To improve NP transport through the mucosa, a permeation enhancer, sodium deoxycholate, was used. The piglet model is an appropriate model for the study of sublingual administration of drugs or biotherapies, as the porcine SL mucosa is closer to the human mucosa than the keratinized SL mucosa of mice. However, the mouse model has been frequently used for preclinical evaluation of SL vaccines, showing suitability for the preliminary evaluation of formulations. 

As the LbL mucoadhesive patch we developed contained CHI, no additional permeation enhancer was used, as this polysaccharide is known to facilitate epithelial transport of therapeutics by disrupting the tight junctions between epithelial cells by translocation of protein zona occludens-1 (ZO-1) [26]. To evaluate the delivery of PLA-NPs from a patch made of a modified CHI, we prepared patches by LbL assembly of HyA and Viscosan^®^ (VIS), a chitosan with increased biodegradability in saliva [10]. As highlighted by Figure 3B (lower panel), the penetration of the NPs in the SL mucosa remains superficial. The NPs did not reach the epithelium and aggregate in the keratinized layer of the mucosa. This result can be explained by the fact that the rapid degradation of the (VIS/HyA)_100_ patch may have impeded efficient delivery of the NPs. Even if, like CHI, the VIS polysaccharide is positively charged, the fast degradation may have impacted the mucoadhesion and drastically reduced the contact time between the LbL patch and the mucosa. Here, we hypothesize that the VIS does not exhibit such permeation ability, explaining the entrapment of the NPs in the external layers of the mucosa without deep penetration.

### 2.5. Bioactivity of Adjuvants after Release from the LbL Patch 

**Figure 4:**
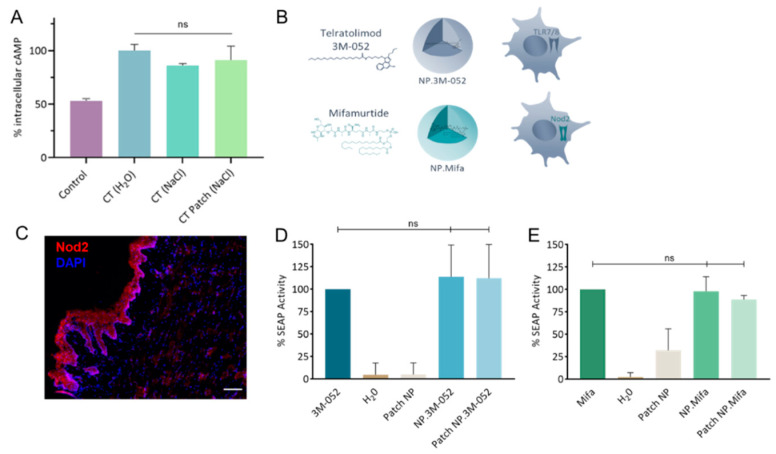
Bioactivity of mucosal adjuvants. (**A**) Quantification of intracellular cAMP produced after 24 h incubation of DC 2.4 cells with CT either formulated as a liquid solution (H_2_O or NaCl) or as an LbL patch in 0.15 mol/L NaCl. The percentage was normalized to the average amount of cAMP produced after contact with CT in H_2_O. (**B**) Schematics of 3M-052 and mifamurtide incorporated in PLA-NP and their respective immune receptors. (**C**) Staining of Nod2 receptors in the SL mucosa of mice. Nod2 receptors (red) and nuclei (DAPI, blue) were stained in naïve mice. Scale bar, 100 µm. (**D**,**E**) Quantification of the SEAP produced after the activation of (**D**) HEK-Blue hTLR7 cells by 3M-052 formulations and (**E**) HEK-Blue hNOD2 cells by mifamurtide formulations, either in their free form, encapsulated in NP, or released from the LbL patch. Data are presented as mean ± SD and were statistically analyzed using one-way ANOVA followed by Tukey’s multiple comparison test (not significant (ns): *p* > 0.05).

The rate of incorporation of CT in the LbL patch was determined to be approximately 78.7 ± 8.8%. The activity of CT after incorporation in the LbL patch was assessed by cAMP quantification (Figure 4A). The bioactivity of CT was maintained after release from the LbL patch. This result illustrates that the incorporation of CT in the patch does not affect its capacity to bind with the GM1 receptor of DCs.

Vaccine adjuvants (hydrophobic molecules) telratolimod (3M-052, agonist of TLR 7/8) and mifamurtide (an agonist of Nod2) were encapsulated in PLA-NPs (Table 1 and Figure 4B). The presence of the Nod2 receptor in the SL mucosa of mice was confirmed (Figure 4C). The encapsulation process did not affect the capacity of the adjuvants to activate their respective immune receptors in HEK-Blue reporter cell lines (Figure 4D,E). Similarly, their incorporation/release from the patch did not reduce their bioactivity. We conclude that the two particulate adjuvants could be used for in vivo evaluation of their impact on the innate response by cytokine/chemokine profiling.

### 2.6. Systemic Inflammatory Response to Primary Sublingual Immunization with a Subunit Vaccine Formulation

Cytokines/chemokines induced by the adjuvants were screened by multiplexing. The cytokine signatures of multiple adjuvants (CT, telratolimod, and mifamurtide) were compared with the cytokine pattern obtained after SL administration of the hapten 1-fluoro-2,4-dinitrobenzene (DNFB). DNFB was shown to induce transient local inflammation in the buccal mucosa (enlarged blood vessels and edema with immune cell infiltrates) [27]. The adjuvants were administered either as a liquid dosage or LbL patch (only for particulate adjuvants 3M-052 and mifamurtide). In order to evaluate the innate immune response to the adjuvanted subunit vaccine, HIV-1 p24 was added to the formulations as a model of a protein antigen, as previously described [20,28]. P24 was coadministered as a liquid dosage, along with CT, or adsorbed on adjuvanted PLA-NP. The administration protocol involved 15 min of anesthesia after liquid deposition to ensure the homogeneity of the results. 

**Figure 5:**
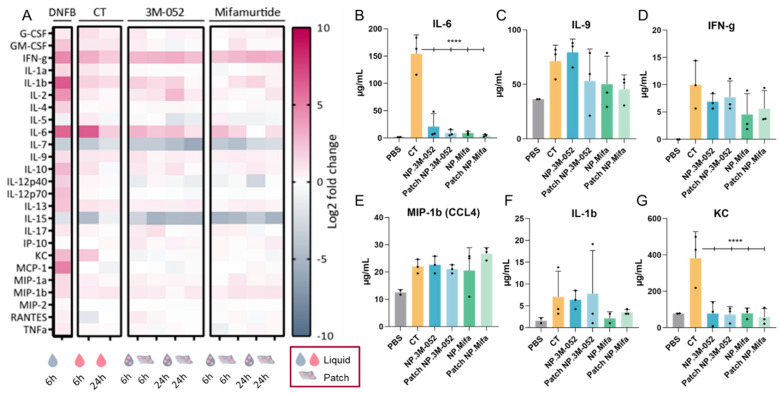
Cytokine/chemokine profiling in serum after SL administration of adjuvanted p24 vaccine formulations. (Mifa: mifamurtide; CT: cholera toxin; NP: nanoparticles; DNFB: 1-fluoro-2,4-dinitrobenzène). (**A**) A total of 25 multiplexed cytokines from the ‘mouse immune panel’ sera were collected either 6 or 24 h after SL administration. Values were calculated as a log2 fold change of the ratio between the mean measured cytokine quantity for 3 mice in each condition over the mean cytokine quantity of control mice (dPBS administration). (**B**–**G**) Quantification of selected cytokines 6 h after SL administration of formulations. Data are presented as mean ± SD and were statistically analyzed using one-way ANOVA followed by Tukey’s multiple comparison test (****: *p* < 0.00001).

The administration of DNFB induced a strong inflammatory response, as evidenced by the remarkable expression of interferon (IFN)-γ, interleukin (IL)-1β, IL-6, keratinocyte chemoattractant (KC), and monocyte chemoattractant protein (MCP)-1 (CCL2) (Figure 5A), as well as the unique expression of IL-10, IL-12p40, IL-12p70, IL-13, and MCP-1 compared to the tested adjuvants. Only DNFB induced a high level of expression of MCP-1, which was involved in the inflammatory process by regulating the infiltration of monocytes and macrophages at the site of inflammation [29]. The inflammation induced by the application of DNFB diverged from the innate immune response induced by an adjuvant, as it led to an overactivation of inflammatory pathways. 

Vaccine adjuvants induce a particular expression of inflammatory cytokines according to several parameters, such as the injection site and the age of individuals. For example, the adjuvant MF59^®^ (AddaVax™), a squalene-based adjuvant, was shown to induce a robust immune response in mice at extremes of ages and induce a specific cytokine signature of IL-5, G-CSF, KC, and MCP-1 [30]. The ability of CT to act as a mucosal adjuvant is partially explained by its ability to induce a Th17 response (IL-17-secreting CD4 T cells), promoting IgA antibody production, as assessed in IL-17A-deficient mice [31]. IgA antibodies are the main players in the mucosal immune response, as they can be secreted in the mucus and block viral infection at the entry gate. Thus, the induction of IL-17 by an adjuvant suggests the potential to induce a mucosal immune response. Lymphocytes from the spleen or the draining lymph node of mice immunized with ovalbumin (OVA) and CT produced IFN-g, IL-4, IL-5, IL-13, IL-10, IL-6, and IL-17A cytokines, suggesting a balanced Th1, Th2, and Th17 immune response after intranasal and intravenous administration [32]. After SL immunization, CT, as well as 3M-052 and mifamurtide, led to IL-17 expression 6 h after vaccine administration, regardless of the form (liquid or patch) (Figure 5A). Similarly to IL-17, the administration of our candidates induced the expression of IFN-γ, IL-1β, IL-6, IL-9, IL-13, and MIP-1β (CCL4), suggesting a cytokine signature close to that of the early inflammatory response induced by CT (Figure 5A–F). However, IL-6 and KC expressions were significantly reduced in NP-formulated 3M-052 and mifamurtide compared to CT (Figure 5A,F). Furthermore, the decrease in IL-7 and IL-15 expression compared to the PBS control is common to CT, 3M-52, and mifamurtide (Figure 5A). 

IFN-γ is a type 2 IFN and a critical cytokine to promote innate and adaptive immunity against pathogens [33]. IFN-γ is predominantly produced by natural killer (NK) cells and natural killer T cells (NKT) and is an activator of macrophages and an inducer of major histocompatibility complex class II (MCH-II) molecule expression. It was recently highlighted that IFN-γ is part of the systemic signature correlating with a robust antibody response to SARS-CoV-2 mRNA vaccines [12]. The sustained systemic expression of IFN-γ during the first 24 h after SL administration of the two tested adjuvants, 3M-052 and mifamurtide, confirmed their potential as SL vaccine adjuvants, with no overactivation of inflammatory chemokines, such as KC or MCP-1, as obtained after DNFB administration (Figure 5A,D). 

As observed for IFN-γ, MIP-1β (CCL4), and IL-1β, the levels of cytokine expression were similar when 3M-052 and mifamurtide were administered as a liquid solution or by the LbL patch. This result suggests an efficient delivery of bioactive adjuvanted NPs through the SL mucosa by the LbL patches and opens the possibility for their use as vaccine delivery systems.

## 3. Materials and Methods

### 3.1. Material 

Medium-molecular-weight chitosan (CHI) was purchased from Sigma-Aldrich. Before its use, CHI was purified by filtering steps and precipitation in water and ethanol, followed by freeze drying, with a final molecular weight of 770 kDa and a degree of deacetylation (DD) of 78%. Viscosan^®^ (VIS), purchased from Flexichem (NAS-081; viscosity, 430 mPas; DD, 49%) was used, owing to its distinct distribution of N-acetylated groups and low DD relative to that of CHI. Sodium hyaluronate (HyA) with molecular weight of 610 kDa (HyA610) was purchased from HTL (Javene, France) and was used as received. 

All reagents used in the present study were of analytical grade and received from commercial sources. Acetone and ethanol were purchased from Carlo Erba (Val-de-Reuil, France). Phosphate-buffered saline (PBS), Dulbecco’s modified eagle medium (DMEM), GlutaMAXTM, DMEM/Ham’s F12 nutrient mixture (1:1), fetal bovine serum (FBS), and penicillin/streptomycin were purchased from Gibco (Thermo Fisher Scientific, Inc., Waltham, MA, USA). Zeocin and blasticidin were obtained from Invivogen (Toulouse, France). Cholera toxin (from *Vibrio cholerae*) was purchased from Sigma-Aldrich (St. Louis, MO, USA). Poly(D,L-lactic acid) (PLA) nanoparticles (PLA-NP), fluorescent (bodipy TR) NPs, telratolimod NPs (NP.3M-052), and mifamurtide (muramyltripeptide mifamurtide, CAS: 83461-56-7) NPs (NP.Mifa) were purchased from Adjuvatis (Lyon, France). HIV-1 p24 antigen was produced and purified by PX’Therapeutics (Grenoble, France).

### 3.2. Adjuvant Formulation: Encapsulation into PLA-NPs

All nanoparticle suspensions were prepared using a surfactant-free nanoprecipitation process. Briefly, PLA polymer was dissolved at 2% (*w*/*v*) in acetone, and this organic solution was added dropwise to an aqueous phase composed of ethanol and 5 mM sodium bicarbonate (Fisher Scientific, Illkirch, France) under 250 rpm stirring. Organic solvents were then removed under reduced pressure at 30 °C with a Rotavapor R-210 (Buchi, France). For the entrapment of either 3M-052, mifamurtide, or red bodipy TR methyl ester (Invitrogen by Fisher Scientific, Waltham, MA, USA) into PLA-NPs, the molecules were added to the organic phase containing the polymer prior to nanoprecipitation. Final PLA-NP suspensions containing 510 µg/mL of telratolimod, 380 µg/mL of mifamurtide, or 3.34 µg/mL of bodipy TR were stored at 4 °C until use. The characteristics of the PLA-NPs are presented in Table 1.

p24 protein was adsorbed either with fluorescent NP-bodipy TR or with NPs loaded with adjuvants. The p24 protein was diluted at a concentration of 600 µg/mL and incubated with NPs for 2 h at RT, with moderate end-overhead stirring. To determine the adsorption yield of p24 protein, the latter was quantified in the supernatant after centrifugation of samples at 10,000 g for 10 min. Quantification was then performed using a micro-BCA^TM^ protein assay kit from Thermo Scientific, and absorbance was measured at 562 nm using a microplate reader. Nanoparticle sizes, polydispersity index, and surface charge were determined using a ZetaSizer Nano ZS Plus (Malvern Instruments, Malvern, UK).

### 3.3. LbL Patch Production and PLA-NP Adsorption

(CHI/HyA) and (VIS/HyA) freestanding patches were produced as described previously [10] using the LbL methodology and a dipping robot (DR-3, Riegler & Kirstein GmbH). Briefly, polyelectrolyte solutions were freshly prepared at 0.2% (*w*/*v*) in a sodium acetate buffer (CH3COONa 0.2 M, CH3COOH 0.2 M, pH = 5.5, RT). The substrates were immersed sequentially in CHI/VIS and HyA solutions with a washing step in sodium acetate buffer between each deposition in polymers solution. A deposition time of 3 min for polyelectrolytes and 2 min for each washing step was used. These immersions were repeated 100 times, and the process was finished by a CHI layer to ensure symmetric mucoadhesion of the produced (CHI/HyA)_100_-CHI patches. For (VIS/HyA) patches, the last layer was VIS. Then, the membranes were left to dry at RT. Finally, membranes were easily detached from their respective underlying substrates by being peeled off with a tweezer. A drop of PLA-NP formulations in sodium acetate buffer was added on the surface of the patches and allowed to dry under air flow for at least 1 h. 

### 3.4. Cell Culture 

HEK-Blue™ hNOD2 and HEK-Blue™ hTLR7 cells were obtained from Invivogen and cultured in DMEM GlutaMAXTM supplemented with 10% (*v*/*v*) FBS, 1% (*v*/*v*) penicillin-streptomycin, 30 µg/mL blasticidin, and 100 µg/mL zeocin. HeLa cells (human epithelial cell line from adenocarcinoma) were obtained from Invivogen and cultured in DMEM GlutaMAXTM supplemented with 10% FBS and 1% (*v*/*v*) penicillin/ streptomycin. Immortalized Ho-1u-1 cells (a human cell line from the floor of mouth squamous cell carcinoma) were obtained from GIMAP (St Etienne, France) and propagated in DMEM/Ham’s F12 nutrient mixture (1:1) supplemented with 10% (*v*/*v*) FBS and 1% (*v*/*v*) penicillin/streptomycin. Immortalized DC2.4 cells (a murine bone-marrow-derived dendritic cell line) were obtained from InvivoGen (Toulouse, France) and cultured in RPMI-1640 medium supplemented with 10% heat-inactivated FBS, 10 mM Hepes, and 50 μM β-mercaptoethanol. All cell lines were maintained in a 37 °C incubator (Heracell 150i, Thermo Scientific) under 5% CO_2_ and 95% humidity.

### 3.5. Cytotoxicity 

Two days prior to cell viability assays, Hela and Ho-1u-1 cells were seeded in a 96-well plate. On the same day, patches (containing NP or not) were cut to size (3 cm^2^ per mL of medium), sterilized under UV light, and incubated overnight at 37 °C in culture medium containing salivary enzymes (lysozyme, α-amylase, and hyaluronidase) at 100 µg/mL. The next day, the culture medium was removed from the 96-well plate and replaced by a medium containing membrane degradation products or PLA-NP diluted in a complete medium. Cells were left to incubate at 37 °C for 24 h. Then, methylthiazolyldiphenyl-tetrazolium bromide (MTT, 0.5 mg/mL) was added to each well, and the resulting plate was incubated for 3 h at 37 °C. A solubilization solution containing 10% (*v*/*v*) of Triton X-100 and HCl (0.1 M) in anhydrous isopropanol was added to cells overnight at RT, protected from light. Absorbance was measured at 570 nm and 690 nm (i-control Infinite^®^ M1000 Pro, Tecan, Männedorf, Switzerland). A negative control was performed with 0.1% (*v*/*v*) SDS, and a positive control was performed using cells alone. Data were determined as the mean of three replicates and three independent experiments. 

### 3.6. Bioactivity of Cholera Toxin after Incorporation into the Mucoadhesive Patch

Prior to the experiments, 0.9 cm² membranes (1 × 0.9 cm) were sterilized by UV light for 20 min. In order to incorporate toxin-derived adjuvant CT, membranes were equilibrated with 1 mM NaCl buffer (NaCl 0.15 M, HEPES 0.02 M, pH 6.5) at RT for 1 h. After equilibration, the excess NaCl was removed, and 12µg of adjuvants was added on top of the membrane. The membranes were left to incubate at 4 °C overnight and rinsed with acetate buffer. Once completely dried, patches were dissolved in 250 µL of water using a homogenizer (30 min, 15 Hz, TissueLyser II, Qiagen, Hilden, Germany). For in vitro intracellular cAMP quantification, DC 2.4 cells were seeded in a 12-well plate at a density of 450,000 cells/well in the appropriate medium. After 24 h, 100 μL of each solution (containing membrane degradation products or toxins) was added to 900 μL of cell medium into the wells. The cells were left in contact with the formulations for 20 h and lysed on ice following the protocol of the cyclic AMP competitive ELISA kit (Thermo Fisher). Data were determined as the mean of two replicates and three independent experiments.

### 3.7. Activation of Innate Immune Receptors by Encapsulated Adjuvants

HEK-Blue™ hTLR7 and HEK-Blue™ hNOD2 were used to quantify the bioactivity of 3M-052 as TLR7 ligand and mifamurtide as NOD2 ligand. Each ligand was evaluated in its free form, encapsulated in PLA-NPs, and as a combination of a patch coated with NP.Mifa or NP.3M-052. In order to monitor the activity of the components on the cells, we used genetically modified cells capable of secreting SEAP upon activation of the NF-κB and AP-1 pathways via activation of TLR7 and Nod2 receptors by the formulated ligands. For the experiment, HEK-Blue™ hTLR7 and HEK-Blue™ hNOD2 were seeded in a 96-well plate at a density of 25,000 cells per well. The cell medium was then replaced with the culture medium containing the formulations, and cells were left in contact with the formulations for 24 h at 37 °C under 5% CO_2_. The SEAP produced by this contact was then quantified by the addition of a developer (HEK-Blue^TM^ Detection, Invivogen), and the absorbance of the samples was measured at 655 nm using a microplate reader (Bio-Rad). Data were determined as the mean of two replicates and three independent experiments. 

### 3.8. Animals

In vivo studies were conducted either on 6-to-8-week-old female CB6F1 mice (Charles River Laboratories, France) at the animal facility PBES of Lyon or on male SKH1 mice (Charles River Laboratories, Saint Germain Nuelles, France) for tomography in the animal facility AniCan at the Cancer Research Center of Lyon (CRCL), France. All animals were maintained in pathogen-free conditions. All of the experiments were performed in accordance with animal welfare regulations for their use for scientific purposes governed by European Directive 2010/63/EU. Protocols were validated by the local Animal Ethics Evaluation Committee (CECCAPP: C2EA-15) and authorized by the French Ministry of Education and Research.

### 3.9. Nod2 Staining in SL Mucosa

Tongues were carefully cut, embedded in OCT compound (Sakura), and stored at −80 °C until cryosection. For NOD2 staining, 6 μm thick sections were prepared using a Cryostat (LEICA), fixed on glass slides with acetone at −20 °C, incubated with a rabbit polyclonal antibody against NOD2 (Novus Biologicals, Bio-Techne Ltd., Abingdon, UK), revealed with goat anti-rabbit secondary antibody conjugated to Alexa Fluor 546 (Invitrogen), and mounted using a Vectashield hard-set mounting medium with Dapi (Vector Laboratories, Eurobio Scientific, Les Ulis, France). Images were captured using an inverted microscope (Nikon Ti-E microscope) equipped with a 10× objective.

### 3.10. In Vivo SL Administration of Formulations

Patches were cut to fit the size of the mouse tongue (2 mm × 6 mm) and sterilized by UV light before PLA-NP addition. Patches or liquid formulations were then administered sublingually (ventral part of the tongue) to anesthetized mice (isoflurane 4%). After administration, a gentle pressure was exerted for few seconds on the dorsal part of the tongue to ensure contact of the patch or the liquid solution with the sublingual mucosa. According to the experiments, the animals were either anesthetized (isoflurane 4 %) for an additional 15 min (Figure 3A and Figure 5) or immediately placed back in their cage (Figure 3B). Once the animals recovered from anesthesia, they were left free to swallow or groom. Water and food were removed for the next 30 min after administration.

### 3.11. Cytokine Quantification by Multiplex Assay

A panel of 20 cytokines/chemokines was simultaneously quantified in each serum sample (collected at 6 or 24 h after SL administration) by Luminex^®^ immunoassay (MILLIPLEX^®^ Mouse cytokine/chemokine panel, Merck) according to the manufacturer’s instructions. Data were determined as the mean of two replicates and three mice per condition.

### 3.12. Statistical Analysis

Statistical analyses were performed using GraphPad Prism version 9.4 software (San Diego, CA, USA). All of the data are presented as the mean ± SD (*n* = 3). Differences between groups were analyzed as described in figure legends. Statistical significance is indicated in the figures. A value of *p* < 0.05 was considered statistically significant.

## 4. Conclusions

The success of mucosal vaccines strongly relies on the efficiency of their vehicle to enhance mucosal penetration of immunogens. Sublingual mucosa for vaccine administration has proven to be a promising route but lacks effective mucoadhesive delivery systems. In this work, we described a mucoadhesive LbL patch for sublingual administration of subunit vaccines consisting of an adjuvanted PLA-NP carrying a viral protein. The PLA-NP was found to be capable of crossing the keratinized layer of the SL mucosa to reach the epithelium. The adjuvants, 3M-052 and mifamurtide, were still bioactive after encapsulation and were able to induce a cytokine/chemokine signature comparable to the gold-standard CT. An evaluation of the antibody humoral and mucosal response after immunization would confirm the full potential of this system to be used as SL vaccine.

## Figures and Tables

**Table 1:** Physicochemical characteristics of PLA-NPs (+/− p24 protein) after encapsulation of telratolimod, mifamurtide, and bodipy TR dye. PDI: polydispersity index.

Encapsulated Molecule +/− p24 Adsorption	Diameter (Z-Average, nm)	PDI	Zeta Potential (mV)
Bodipy TR	176.3 ± 1.8	0.051 ± 0.022	−59.1 ± 1.1
Bodipy TR + p24	210.5 ± 5.5	0.111 ± 0.035	−51.6 ± 1.1
Telratolimod	134.6 ± 0.1	0.127 ± 0.083	−59.9 ± 2.4
Telratolimod + p24	155.5 ± 0.8	0.175 ± 0.036	−45.4 ± 0.8
Mifamurtide	158.7 ± 0.7	0.053 ± 0.007	−60.0 ± 0.5
Mifamurtide + p24	165.0 ± 0.4	0.057 ± 0.010	−53.8 ± 1.6

## Data Availability

Not applicable.
